# Discriminative Performance of Anthropometric and Body Composition Measures for Identifying CONUT-Defined Nutritional Risk in Hospitalized Older Adults: A Cross-Sectional Study

**DOI:** 10.3390/nu18203316

**Published:** 2026-10-09

**Authors:** Justyna Nowak, Marzena Jabczyk, Michał Skrzypek, Michał Górski, Barbara Zubelewicz-Szkodzińska

**Affiliations:** 1 Department of Cardiovascular Disease Prevention, Department of Metabolic Disease Prevention, Faculty of Public Health in Bytom, Medical University of Silesia, 41-902 Bytom, Poland; 2 Department of Nutrition-Related Disease Prevention, Department of Metabolic Disease Prevention, Faculty of Public Health in Bytom, Medical University of Silesia, 41-902 Bytom, Poland; 3 Department of Biostatistics, Faculty of Public Health in Bytom, Medical University of Silesia, 41-902 Bytom, Poland; mskrzypek@sum.edu.pl; 4 Department of Psychiatry, Department of Neurology, Faculty of Health Sciences in Katowice, Medical University of Silesia in Katowice, 40-635 Katowice, Poland; 5 Department of Endocrinology, District Hospital, 41-940 Piekary Slaskie, Poland

**Keywords:** aged, anthropometry, body composition, nutrition assessment, nutritional status, CONUT

## Abstract

**Background/Objective:** The Controlling Nutritional Status (CONUT) score is a laboratory-based tool for identifying nutritional risk. This study examined associations of anthropometric and bioimpedance-derived body composition measures with CONUT and their ability to discriminate CONUT-defined nutritional risk in hospitalized older adults. **Methods:** This cross-sectional study included 164 hospitalized adults aged >60 years. Anthropometric measurements, derived indices, and bioimpedance-derived body composition parameters were assessed, and CONUT was calculated from routine laboratory data. **Results:** CONUT-defined nutritional risk was present in 43.3% of participants. Participants with nutritional risk had lower body weight, waist, mid-upper arm and calf circumferences, fat mass, body mass index (BMI), and body adiposity index (BAI) (all *p* < 0.05). Correlations with the CONUT score were weak; the largest absolute correlations were observed for fat mass in kilograms and fat mass percentage (both ρ = −0.25). In the fully adjusted model, mid-upper arm circumference (OR = 0.900; 95% CI 0.825–0.982), fat mass in kilograms (OR = 0.955; 95% CI 0.921–0.991), and fat mass percentage (OR = 0.928; 95% CI 0.887–0.972) showed nominally significant associations with lower odds of CONUT-defined nutritional risk. After FDR adjustment, only fat mass percentage remained statistically significant (q = 0.024). Discriminatory performance was modest; the highest areas under the curve (AUCs) were 0.655 for fat mass percentage, 0.647 for fat mass in kilograms, and 0.638 for mid-upper arm circumference. **Conclusions:** Anthropometric and body composition measures showed limited ability to discriminate CONUT-defined nutritional risk and should not be considered substitutes for CONUT. Among the evaluated measures, fat mass percentage showed the most robust association with CONUT-defined nutritional risk. More complex derived indices did not provide additional discriminatory value.

## 1. Introduction

Malnutrition is a common and clinically important problem among hospitalized older adults. Age-related anorexia, multimorbidity, acute and chronic diseases, and impaired functional status may reduce food intake. In addition, disease-related inflammation and catabolism may accelerate the deterioration of nutritional status. Malnutrition in older patients is associated with a higher risk of infection, longer hospital stay, delayed recovery, and increased mortality. It is also linked to functional decline, frailty, and sarcopenia. Consequently, the European Society for Clinical Nutrition and Metabolism recommends routine screening for malnutrition in all older persons. However, reported prevalence estimates vary considerably depending on the healthcare setting, patient characteristics, and the screening or diagnostic criteria used [1,2,3].

Nutritional screening and diagnostic assessment are related but distinct stages of care. Screening tools are intended to identify individuals who may require further evaluation and do not, by themselves, confirm a diagnosis of malnutrition. The Global Leadership Initiative on Malnutrition (GLIM) framework uses a two-step approach consisting of initial screening for malnutrition risk followed by diagnostic assessment based on phenotypic and etiologic criteria. A diagnosis requires at least one phenotypic and one etiologic criterion [4]. Different screening tools may yield different classifications and show variable ability to identify GLIM-defined malnutrition [4,5]. Therefore, the result of a single screening tool should be interpreted as an indication of nutritional risk defined by that tool rather than as a confirmed diagnosis of malnutrition.

The Controlling Nutritional Status (CONUT) score is a laboratory-based screening index calculated from serum albumin, total cholesterol, and total lymphocyte count. It was originally developed to enable automatic daily screening of hospitalized patients using routinely available laboratory data [6]. Because it does not require a patient interview or bedside anthropometric measurements, CONUT may be useful in geriatric care [7]. However, the laboratory variables included in the score are also influenced by disease and treatment [6]. Serum albumin is particularly affected by inflammation and changes in capillary permeability and should not be interpreted as a direct marker of body protein stores or muscle mass [8]. Therefore, CONUT should be interpreted as a laboratory-based indicator of nutritional risk rather than as confirmation of malnutrition. It does not directly assess anthropometric characteristics or body composition.

Anthropometric measurements may complement laboratory-based nutritional risk assessment by providing information on body size and regional body dimensions [7,9,10]. These measurements are simple, inexpensive, and noninvasive and can be used in the nutritional assessment of older adults [9,10]. Body mass index (BMI) and arm, calf, waist, and hip circumferences describe different aspects of body size, while bioelectrical impedance analysis provides estimates of fat mass, fat-free mass, and muscle mass [7,9]. Derived indices, such as the waist-to-hip ratio, waist-to-height ratio, body adiposity index, body roundness index, and abdominal volume index, have also been examined in nutritional assessments of older populations [9]. Studies have proposed sex-specific cut-off values, but direct comparisons are limited by differences in the nutritional reference methods used [9,10].

Few studies have examined CONUT together with anthropometric and body composition measures in hospitalized older adults [7,11]. Lo Buglio et al. found that higher CONUT categories were associated with lower BMI and selected body circumferences, lower muscle mass percentage, and higher fat-mass percentage [7]. Sun et al. reported that a high CONUT category defined using sex-specific cut-offs was independently associated with sarcopenia, although correlations with the relative skeletal muscle mass index and handgrip strength were weak or absent [11]. In a small pilot study of non-hospitalized older and long living individuals, Aiello et al. reported discordant findings obtained using the Mini Nutritional Assessment (MNA), bioelectrical impedance analysis and bioelectrical impedance vector analysis (BIA/BIVA), Geriatric Nutritional Risk Index (GNRI) and CONUT score, particularly among the oldest participants [12]. These findings indicate that CONUT, anthropometry, and body composition provide related but distinct information. However, none of these studies jointly evaluated conventional anthropometric measurements, bioimpedance-derived body composition, and a broad range of derived anthropometric indices in a single hospitalized geriatric population [7,11,12].

In a previous study by our group, several anthropometric measures and indices showed strong discrimination of GNRI-defined nutritional risk [13]. However, because current body weight is part of the GNRI formula, associations with body weight, BMI, and indices that include BMI may partly reflect shared mathematical components. By contrast, CONUT contains no anthropometric variable, allowing associations with anthropometric characteristics and body composition to be examined without direct mathematical overlap [6,13].

Therefore, this study aimed to examine the associations between the CONUT score and anthropometric measurements, derived anthropometric indices, and bioimpedance-derived body composition parameters in hospitalized older adults. We also assessed the ability of these measures to discriminate CONUT-defined nutritional risk.

## 2. Materials and Methods

### 2.1. Ethics Approval

The study was conducted in accordance with the ethical principles of the Declaration of Helsinki and was approved by the Bioethics Committee of the Medical University of Silesia in Katowice (initial approval no. KNW/0022/KB1/53/14; continuation approval no. BNW/NWN/0022/KB1/53/III/14/16/25). Prior to enrolment, all participants received detailed information regarding the purpose and procedures of the study and provided informed consent. Participation was voluntary, and all participants were informed of their right to withdraw from the study at any time without any consequences for their medical care. No additional diagnostic or laboratory procedures were performed specifically for the purposes of the study. Laboratory data were extracted from the patients’ medical records and included only routine tests performed as part of standard in-hospital clinical care. All collected data were deidentified before analysis, stored securely, and reported exclusively in aggregate form, thereby preventing the identification of individual participants.

### 2.2. Study Design and Participants

A cross sectional study was conducted among patients aged >60 years hospitalized in the Geriatric Department. The present analysis used data collected within an ongoing geriatric research framework.

**Figure 1:**
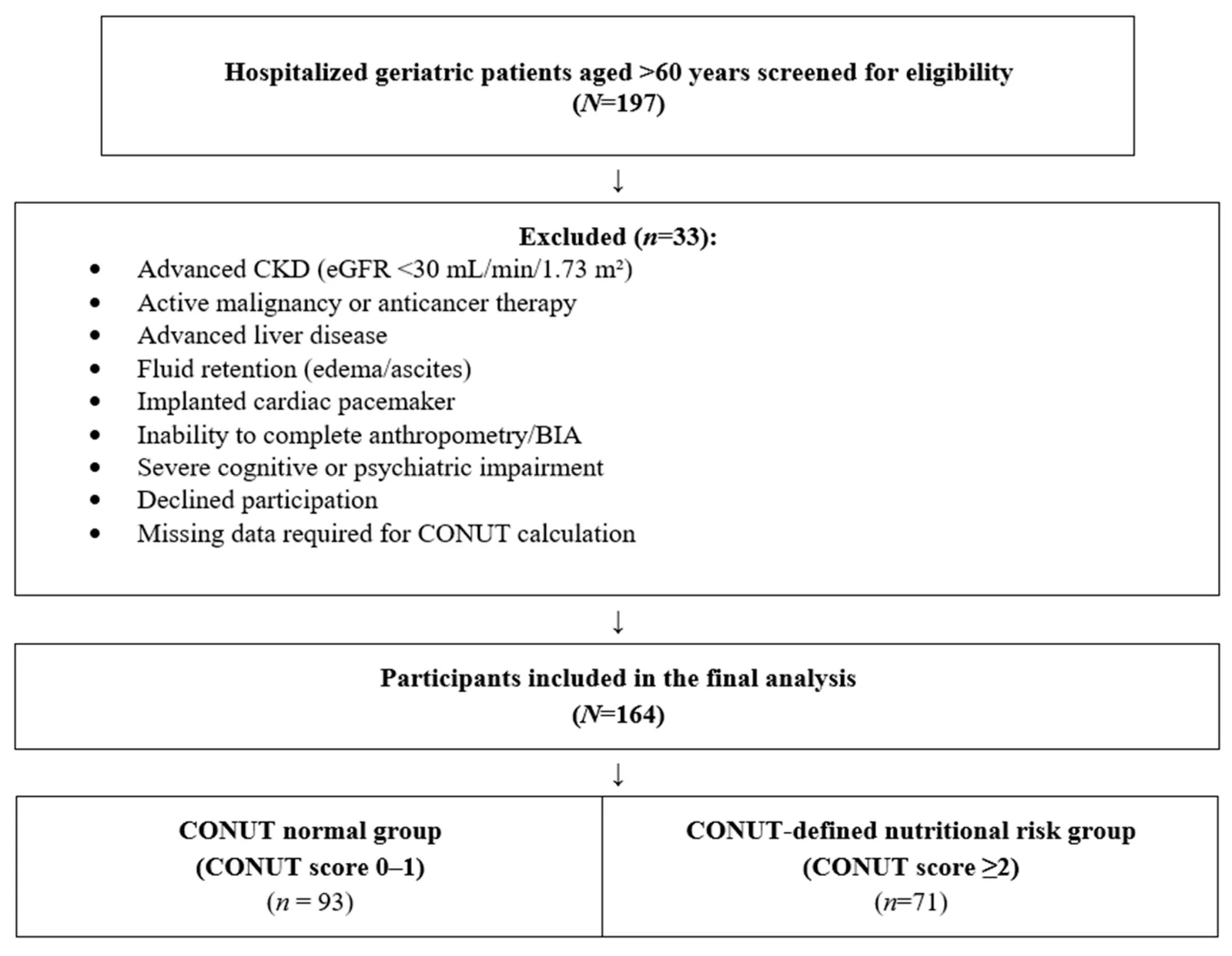
Flow diagram of participant selection.

Clinical information, including medical history, comorbidities, current pharmacotherapy, and laboratory test results, was obtained from the patients’ medical records. Only clinical and laboratory data collected as part of routine in-hospital care were used in the present study, and no additional laboratory testing was performed for research purposes. Anthropometric measurements were performed during hospitalization according to standardized procedures as part of routine geriatric assessment. The exclusion criteria were active malignancy or ongoing anticancer treatment; advanced chronic liver disease, including liver cirrhosis; stage 4 or higher chronic kidney disease, defined as an estimated glomerular filtration rate (eGFR) <30 mL/min/1.73 m^2^; clinical signs of pathological fluid retention, including peripheral edema or ascites; an implanted cardiac pacemaker; substantial mobility limitations preventing reliable anthropometric measurements or body composition assessment using BIA; severe cognitive or psychiatric impairment precluding informed participation; and lack of informed consent. Use of medications potentially affecting fluid balance, including diuretics, was not considered an exclusion criterion. For the purposes of the present analysis, patients with missing serum albumin, total cholesterol, or total lymphocyte count measurements were additionally excluded because these parameters are required to calculate the Controlling Nutritional Status (CONUT) score. As no additional laboratory tests were performed for research purposes, participants with incomplete routinely collected laboratory data required for CONUT calculation could not be included in the analysis. Each participant was included only once. Patients rehospitalized during the study period were neither reassessed nor re-enrolled. The participant selection process is presented in Figure 1.

### 2.3. Clinical, Anthropometric, and Body Composition Assessment

Clinical data, including age, sex, medical history, comorbidities, and current pharmacotherapy, were obtained from the patients’ medical records. Information regarding rehospitalization during the study period was obtained through direct patient interview to prevent repeated enrolment.

All anthropometric measurements were performed once by a study investigator with appropriate expertise in anthropometric assessment, using standardized procedures and the same measuring instruments. Anthropometric and body composition assessments were performed in the morning after an overnight fast and after voiding, with participants barefoot and wearing light clothing. Body weight, height, waist circumference, hip circumference, mid-upper arm circumference, and calf circumference were measured according to standardized procedures. Body weight was measured to the nearest 0.1 kg using a Tanita BC-420 S MA analyzer (Tanita Corporation, Tokyo, Japan), and height was measured to the nearest 5 mm using a Tanita HR-100 stadiometer. Waist, hip, mid-upper arm, and calf circumferences were measured to the nearest 1 mm using a Seca 203 anthropometric measuring tape (Seca GmbH & Co. KG, Hamburg, Germany).

Body height was measured in the standing position, with participants barefoot, feet placed together, arms relaxed alongside the body, and the head maintained in a neutral position while looking straight ahead. Waist circumference was measured in the area between the lower margin of the rib cage and the iliac crest, with the measuring tape positioned horizontally around the trunk; the measurement was taken at the end of a gentle expiration [14]. Hip circumference was measured at the level of the greatest posterior protrusion of the buttocks, with the tape maintained horizontally [14]. Mid-upper arm circumference was measured on the non-dominant upper limb, with the arm relaxed alongside the body, at the midpoint between the acromion process of the scapula and the olecranon process of the ulna [15]. Calf circumference was measured on the left lower limb at the point of maximum calf girth, with participants standing upright, feet slightly apart, and body weight evenly distributed between both legs [16]. Before calf circumference assessment, the lower limbs were inspected for edema.

Body composition was assessed using a Tanita BC-420 S MA Body Composition Analyzer (Tanita Corporation, Tokyo, Japan), a single-frequency tetrapolar foot-to-foot bioelectrical impedance analyzer operating at 50 kHz and 90 μA [17]. Measurements were performed in the morning after an overnight fast and after voiding, with participants barefoot and wearing light clothing. During the measurement, participants stood upright and still on the analyzer platform, with both feet positioned on the foot electrodes in accordance with the manufacturer’s instructions [17]. The recorded BIA-derived parameters included fat mass expressed in kilograms and as a percentage of body weight, fat-free mass and muscle mass. Previous comparison of the BC-420MA with dual-energy X-ray absorptiometry (DXA) showed strong correlations for fat mass in kilograms, but also indicated that BIA-derived estimates should not be interpreted as directly interchangeable with DXA measurements [18].

### 2.4. Anthropometric and Adiposity Indices

Anthropometric and adiposity indices were calculated from the obtained anthropometric and laboratory measurements according to the formulas presented in Table 1.

### 2.5. Laboratory Measurements

Laboratory data obtained on the day of hospital admission were retrieved exclusively from the patients’ medical records. All tests were performed after an overnight fast in accordance with the hospital’s standard clinical procedures. No additional blood samples were collected and no laboratory tests were performed specifically for research purposes. The parameters included in the analysis were serum albumin concentration (g/dL), total lymphocyte count (cells/mm^3^), total cholesterol, low-density lipoprotein (LDL) cholesterol, high-density lipoprotein (HDL) cholesterol, and triglyceride concentrations (mmol/L), C-reactive protein (CRP) concentration (mg/L), and estimated glomerular filtration rate (eGFR; mL/min/1.73 m^2^). For calculation of the CONUT score, total cholesterol concentrations reported in mmol/L were converted to mg/dL by multiplying the values by 38.67.

### 2.6. Nutritional Risk Assessment Using the CONUT Score

The CONUT score was calculated based on serum albumin concentration, total cholesterol concentration, and total lymphocyte count, as presented in Table 2. Participants were classified as having a normal CONUT score (0–1 points), mild nutritional risk (2–4 points), moderate nutritional risk (5–8 points), or severe nutritional risk (9–12 points). For the analyses, the CONUT score was dichotomized into two groups: a CONUT-normal group (0–1 points) and a CONUT-defined nutritional risk group (≥2 points). Owing to the small number of participants in the moderate risk category and the absence of participants in the severe risk category, the mild- and moderate risk categories were combined.

### 2.7. Sample Size Estimation

The minimum required sample size for the receiver operating characteristic (ROC) analysis was calculated assuming an area under the curve (AUC) of 0.70, a significance level of 0.05, a statistical power of 0.80, and a 1:1 ratio of participants with and without CONUT-defined nutritional risk. The estimated minimum sample size was 31 participants per group (62 participants in total). The final analytical sample met these requirements and included 71 participants with CONUT-defined nutritional risk and 93 participants with a normal CONUT score. Calculations were performed using the pROC package [27] in R software, version 4.6.1 (R Foundation for Statistical Computing, Vienna, Austria) [28].

For the multivariable logistic regression analyses, the number of explanatory variables was limited according to the number of outcome events. With 71 participants classified as having CONUT-defined nutritional risk, the fully adjusted models remained within the conventional criterion of approximately 10 outcome events per explanatory variable.

### 2.8. Statistical Analysis

Statistical analyses were performed using R software, version 4.6.1 (R Foundation for Statistical Computing, Vienna, Austria). The distribution of continuous variables was assessed using the Shapiro–Wilk test and visual inspection of distribution plots. Continuous variables are presented as mean and standard deviation for approximately normally distributed data or as median and interquartile range for non-normally distributed data. Categorical variables are reported as numbers and percentages. Missing data were handled using analysis-specific complete case exclusion; participants with missing values for any variable required for a given analysis were excluded from that analysis.

Comparisons between the CONUT-normal group and the CONUT-defined nutritional risk group were performed using Welch’s two-sample t test for approximately normally distributed continuous variables and the Mann–Whitney U test for variables with non-normal distributions. Categorical variables were compared using the chi-square test or Fisher’s exact test when expected cell counts were small.

Associations between the total CONUT score and anthropometric measurements, body composition parameters, and derived anthropometric indices were assessed using Spearman’s rank correlation coefficient.

Receiver operating characteristic curves were constructed to evaluate the ability of individual anthropometric measurements, body composition parameters, and derived anthropometric indices to discriminate participants with CONUT-defined nutritional risk from those with a normal CONUT score. Discriminatory performance was expressed as the area under the curve with 95% confidence intervals. Internal validation of discriminatory performance was performed using bootstrap resampling with 200 resamples, evaluated by the *C*-index with 95% confidence intervals. Exploratory cut-off values for individual parameters were identified using the Youden index. Sensitivity, specificity, positive predictive value, and negative predictive value were calculated for the selected cut-off points.

Binary logistic regression was used to evaluate the association between each anthropometric measurement, body composition parameter, or derived anthropometric index and CONUT-defined nutritional risk. The results are presented as odds ratios with 95% confidence intervals. Owing to the substantial correlations among the examined anthropometric and body composition parameters, each parameter was entered into a separate regression model. Model 1 was adjusted for age and sex; Model 2 for age, sex, and statin use; Model 3 for age, sex, and C-reactive protein concentration; and Model 4 for age, sex, statin use, and C-reactive protein concentration. To account for multiple hypothesis testing, *p*-values were adjusted separately for each model using the Benjamini–Hochberg procedure to control the false discovery rate (FDR). Both nominal *p*-values and FDR-adjusted *p*-values (q values) are reported. Variables used directly to calculate the CONUT score, namely serum albumin, total cholesterol, and total lymphocyte count, were not entered as independent predictors. All statistical tests were two-sided, and a *p* value < 0.05 was considered statistically significant.

## 3. Results

Demographic, clinical, and laboratory characteristics of the total study population and according to CONUT category are presented in Table 3. Among the 164 participants included in the analysis, 93 (56.7%) had a normal CONUT score, 63 (38.4%) had mild nutritional risk, and 8 (4.9%) had moderate nutritional risk; no participants were classified as having severe nutritional risk. Accordingly, 71 participants (43.3%) were included in the CONUT-defined nutritional risk group. Compared with the CONUT-normal group, participants in the CONUT-defined nutritional risk group were older (80.2 ± 6.3 vs. 78.1 ± 6.2 years, *p* = 0.034), whereas the proportion of women did not differ significantly between the groups. Atrial fibrillation was more common in the CONUT-defined nutritional risk group (28.2% vs. 15.1%, *p* = 0.040), while arterial hypertension (73.2% vs. 88.2%, *p* = 0.014) and diabetes mellitus (23.9% vs. 40.9%, *p* = 0.023) were less prevalent. Anemia was also significantly more frequent in participants with CONUT-defined nutritional risk (39.4% vs. 12.9%, *p* < 0.001).

Anthropometric measurements, body composition parameters, and derived anthropometric indices according to CONUT category are presented in Table 4. Compared with the CONUT-normal group, participants with CONUT-defined nutritional risk had lower body weight 64.0 (54.3–74.7) vs. 69.2 (61.2–79.4) kg, *p* = 0.014; waist circumference 101.0 (95.0–109.0) vs. 105.0 (99.0–114.0) cm, *p* = 0.013; mid-upper arm circumference 27.27 ± 4.25 vs. 29.37 ± 4.06 cm, *p* = 0.002; and calf circumference 33.82 ± 3.70 vs. 35.35 ± 4.05 cm, *p* = 0.012. Fat mass was also lower, both in kg 20.1 (13.3–26.4) vs. 23.9 (19.3–31.6) kg, *p* = 0.001 and as a percentage of body weight 30.55 (24.10–37.20) vs. 36.05 (29.20–42.85)%, *p* < 0.001; whereas fat-free mass and muscle mass did not differ significantly between the groups. Among the derived anthropometric indices, BMI 25.9 (22.8–29.8) vs. 27.7 (25.5–31.7) kg/m^2^, *p* = 0.009 and BAI 34.37 (29.34–38.59) vs. 36.40 (31.34–41.16)%, *p* = 0.042 were lower in the CONUT-defined nutritional risk group.

As shown in Table 5, the CONUT score was weakly but significantly negatively correlated with body weight (ρ = −0.21; *p* = 0.008), mid-upper arm circumference (ρ = −0.19; *p* = 0.017), calf circumference (ρ = −0.17; *p* = 0.034), fat mass (kg: ρ = −0.25; *p* = 0.002; %: ρ = −0.25; *p* = 0.001), and BMI (ρ = −0.18; *p* = 0.021). No significant correlations were observed between the CONUT score and the remaining anthropometric indices.

Associations of anthropometric measurements, body composition parameters, and derived anthropometric indices with CONUT-defined nutritional risk in multivariable logistic regression models are presented in Table 6. In the fully adjusted model accounting for age, sex, statin use, and CRP, mid-upper arm circumference (OR = 0.900, 95% CI 0.825–0.982, *p* = 0.0177, q = 0.094), fat mass in kilograms (OR = 0.955, 95% CI 0.921–0.991, *p* = 0.0136, q = 0.094), and fat mass percentage (OR = 0.928, 95% CI 0.887–0.972, *p* = 0.0015, q = 0.024), showed nominally significant associations with lower odds of CONUT-defined nutritional risk. After adjustment for multiple testing using the Benjamini–Hochberg procedure, only fat mass percentage remained statistically significant. Calf circumference showed nominally significant associations in Models 1 and 2 (*p* = 0.0443 and *p* = 0.0481, respectively), but these associations did not remain significant after FDR adjustment (q = 0.177 and q = 0.192, respectively). No other anthropometric measurement, body composition parameter, or derived anthropometric index remained statistically significant after FDR adjustment.

Discriminative performance of anthropometric measurements, body composition parameters, and derived anthropometric indices for identifying CONUT-defined nutritional risk is presented in Table 7. The highest AUC values were observed for fat mass percentage AUC = 0.655 (95% CI 0.570–0.741), cutoff 38.5%; fat mass in kilograms AUC = 0.647 (95% CI 0.560–0.734), cutoff 21.4 kg and mid-upper arm circumference AUC = 0.638 (95% CI 0.552–0.723), cutoff 29 cm. Boot-strap-validated c-indices were similar for these parameters (0.651, 0.641, and 0.642, respectively).

## 4. Discussion

The present study evaluated the associations between the CONUT score and anthropometric measurements, derived anthropometric indices, and bioimpedance-derived body composition parameters in hospitalized older adults, as well as the ability of these measures to discriminate CONUT-defined nutritional risk. Participants with CONUT-defined nutritional risk generally had lower values of several anthropometric measures (body weight, waist circumference, and mid-upper arm circumference), body composition parameters (fat mass expressed in kilograms and as a percentage of body weight), and derived indices (BMI and BAI). Participants with CONUT-defined nutritional risk were slightly older than those with a normal CONUT score. This finding is consistent with previous evidence showing that advancing age is associated with an increased risk of malnutrition in older adults, likely reflecting the accumulation of age-related physiological, functional, and disease-related factors [29,30]. However, the overall associations with the CONUT score were weak. Fat mass percentage showed the most robust association with CONUT-defined nutritional risk and remained statistically significant after false discovery rate (FDR) adjustment. Mid-upper arm circumference and fat mass in kilograms showed nominally significant associations with lower odds of CONUT-defined nutritional risk in the fully adjusted model, but these associations did not remain statistically significant after FDR adjustment. Muscle mass was not significantly associated with the CONUT score. The discriminatory performance of individual anthropometric and body composition measures was modest. Overall, the observed associations with the CONUT score were weak, and only fat mass percentage remained significantly associated with CONUT-defined nutritional risk after FDR adjustment.

An important practical feature of the CONUT score is that it is calculated entirely from routinely available laboratory parameters and does not require anthropometric measurements or a patient interview [6,7]. At the same time, its components are influenced by factors other than nutritional status, particularly systemic inflammation and disease-related changes, and CONUT does not directly characterize body size or body composition [6,8]. Therefore, evaluating whether simple anthropometric and body composition measures correspond to CONUT-defined nutritional risk is clinically relevant, particularly in hospitalized older adults. Importantly, CONUT was not used as a diagnostic reference standard for malnutrition. Accordingly, the AUC values reported in this study reflect the ability of individual anthropometric and body composition measures to discriminate CONUT-defined nutritional risk, rather than their diagnostic accuracy for malnutrition. In our previous GNRI-based analysis, anthropometric measures showed substantially stronger associations with GNRI-defined nutritional risk [13]. BMI and body fat in kilograms were strongly correlated with the GNRI, and BMI showed excellent discrimination of GNRI-defined nutritional risk, with an AUC of 0.93 [13]. These findings contrast with the weak correlations and modest discriminatory performance observed in the present CONUT-based analysis. This difference should, however, be interpreted in the context of the construction of the two indices. Current body weight is directly incorporated into the GNRI formula, which may contribute to the strong associations of the GNRI with body weight, BMI, and other weight-dependent anthropometric measures. In contrast, the CONUT score contains no anthropometric component [6,13]. Accordingly, the weaker associations observed in the present study should be interpreted in light of the different components used to construct the GNRI and CONUT.

The findings of the present study are partially consistent with previous studies examining the relationships between CONUT, anthropometry, and body composition. In 361 hospitalized adults aged ≥65 years, Lo Buglio et al. [7] found that higher CONUT categories were associated with lower BMI and smaller arm and calf circumferences, which is consistent with the anthropometric differences observed in our study. However, the findings regarding body composition were less concordant. Lo Buglio et al. reported lower skeletal muscle mass and fat-free mass, together with a higher percentage of fat mass, in patients with higher CONUT scores [7], whereas in our study fat mass was lower in the CONUT-risk group and muscle mass did not differ significantly between groups. The discrepancy in muscle-related findings may partly reflect methodological differences in body composition assessment, including the use of different BIA devices and device-specific prediction algorithms [7,31]. Differences in the populations studied and in the distribution of CONUT severity may also have contributed to these divergent findings. Sun et al. [11] similarly reported an independent association between a higher CONUT score and sarcopenia in hospitalized older adults. Nevertheless, BMI, relative skeletal muscle mass index, and handgrip strength did not differ significantly between patients with high and low CONUT scores within the sarcopenia group, and most associations between CONUT and individual sarcopenia-related parameters were weak or nonsignificant [11]. These observations are consistent with the generally weak associations between CONUT and individual anthropometric and body composition measures found in our study. Finally, Aiello et al. [12] demonstrated discordance between nutritional assessment methods in older adults: MNA and BIA/BIVA suggested nutritional impairment in some of the oldest participants, whereas CONUT and GNRI indicated normal or relatively low nutritional risk. These findings support the view that laboratory-based nutritional indices and anthropometric or body composition assessments provide complementary rather than interchangeable information [12].

Simple anthropometric measurements such as mid-upper arm and calf circumferences may provide useful information on nutritional status and tissue reserves in older adults, particularly when conventional or instrument-based assessments are difficult to perform [10,32]. This practical role is also reflected in current expert recommendations. The GLIM guidance supports the use of anthropometric measures, including calf circumference and mid-arm muscle circumference, for the assessment of reduced muscle mass when methods such as BIA, dual-energy X-ray absorptiometry (DXA), computed tomography (CT), magnetic resonance imaging (MRI), or ultrasound are unavailable or not feasible [4,33]. Similarly, the European Working Group on Sarcopenia in Older People 2 (EWGSOP2) consensus indicates that, although anthropometry is not considered a preferred direct measure of muscle mass, calf circumference may be used as a diagnostic proxy in older adults when other methods for assessing muscle quantity are not available [31]. Simple circumference measurements may therefore be useful in settings with limited access to advanced body composition techniques, although they should be regarded as surrogate rather than equivalent measures. Their interpretation nevertheless requires caution, particularly in individuals with greater adiposity, because arm circumference reflects both muscle mass and subcutaneous adipose tissue [34].

In the present study, both mid-upper arm and calf circumferences were lower in participants with CONUT-defined nutritional risk and showed weak inverse correlations with the CONUT score. However, mid-upper arm circumference showed a nominally significant association with lower odds of CONUT-defined nutritional risk in the fully adjusted model, although this association did not remain statistically significant after FDR adjustment. Calf circumference showed nominally significant associations in Models 1 and 2, but these associations also did not remain significant after FDR adjustment. A mid-upper arm circumference cut-off of 29 cm showed only limited discriminatory performance for CONUT-defined nutritional risk (AUC = 0.638), despite relatively good sensitivity (73%) but low specificity (51%). These findings suggest that mid-upper arm circumference may provide useful complementary information in the nutritional assessment of older adults, but its limited discriminatory ability and lack of statistical significance after FDR adjustment do not support its use as a standalone surrogate for CONUT-defined nutritional risk. The absence of a significant association between CONUT and muscle mass in the present study is not unexpected, as none of the components of the CONUT score directly assess muscle quantity [6]. In particular, serum albumin is influenced by inflammation and should not be interpreted as a proxy measure of total body protein or muscle mass [8]. Une et al. found no significant association between CONUT and dichotomously defined CT-based sarcopenia, despite an inverse relationship between CONUT and skeletal muscle index in oncology patients [35].

Fat mass showed the strongest overall pattern of association with CONUT-defined nutritional risk in the present study. Both fat mass in kilograms and fat mass percentage showed nominally significant associations in the fully adjusted model; however, after FDR correction, only fat mass percentage remained statistically significant. Both parameters also demonstrated the highest, although still modest, discriminatory performance among the measures examined. The lower fat mass observed in participants with CONUT-defined nutritional risk may reflect lower energy reserves. However, the cross sectional design does not establish whether the lower fat mass resulted from preceding nutritional deterioration. Inadequate energy and protein intake can result in loss of both fat and fat-free mass, and nutritional deterioration may therefore affect multiple body compartments [36]. However, changes in body composition are heterogeneous, particularly in disease-related malnutrition, where reduced food intake or assimilation, inflammation, and muscle- and fat-specific catabolic mechanisms may contribute to the malnourished phenotype to varying degrees [3,37]. In contrast, none of the derived anthropometric indices showed statistically significant associations with CONUT-defined nutritional risk after FDR adjustment. More complex derived indices did not demonstrate superior discriminatory performance or more robust associations than simpler anthropometric and body composition measures in this population. From a practical perspective, the limited discriminatory performance observed in the present study indicates that simple anthropometric measures should not be used as substitutes for CONUT-based classification.

Although CONUT is a practical laboratory-based screening tool that relies on routinely available laboratory parameters [6], its components are not specific to malnutrition. Serum albumin concentration is strongly influenced by inflammation and illness [8], whereas total cholesterol may also be affected by lipid lowering therapy, an issue specifically highlighted in older adults by Göçer et al. [38]. These considerations provided a biological rationale for accounting for CRP concentration and statin use as potential confounding factors in the analyses performed in the present study [8,38].

Nutritional risk identified by screening should prompt further assessment of nutritional status rather than be regarded as a diagnosis of malnutrition itself [1,4,5]. Overall, the weak associations with the CONUT score and limited discriminatory performance indicate that anthropometric measurements alone cannot reliably reproduce CONUT-defined nutritional risk. Accordingly, anthropometric measurements should not be considered substitutes for either CONUT-based classification or formal diagnostic assessment of malnutrition.

### Study Limitations

Several limitations of this study should be acknowledged. First, the relatively small sample size and single-center design may limit the generalizability of the findings. In addition, the exclusion of patients with clinically evident edema or ascites, implanted cardiac pacemakers, and advanced chronic kidney disease, although necessary for BIA assessment, may have reduced the representation of frailer hospitalized older adults and further limits generalizability to the broader geriatric inpatient population. Second, the cross-sectional design precludes conclusions regarding temporal or causal relationships between CONUT-defined nutritional risk and anthropometric or body composition characteristics. Third, most participants with CONUT-defined nutritional risk had mild risk, with only a small number of moderate-risk cases and no severe-risk cases, limiting evaluation across the full spectrum of CONUT severity. Fourth, CONUT was used as a laboratory-based nutritional risk screening tool rather than as a diagnostic reference standard for malnutrition, and no independent diagnostic assessment based on GLIM criteria was performed. Accordingly, the reported AUC values should be interpreted as reflecting discrimination of CONUT-defined nutritional risk rather than diagnostic accuracy for malnutrition. Fifth, body composition was estimated using BIA rather than reference techniques such as DXA, CT, or MRI. The derived values are based on device-specific proprietary prediction equations and may be influenced by hydration status. Although measurements were standardized after an overnight fast and voiding and patients with clinically evident fluid retention were excluded, subclinical alterations in hydration status, including those potentially related to medications affecting fluid balance, could not be excluded. Moreover, separate resistance and reactance measurements required for conventional BIVA were unavailable, precluding an independent assessment of hydration status. Sixth, replicate anthropometric measurements were not included in the study protocol; therefore, study-specific measurement reproducibility could not be assessed. Finally, the sample size calculation assumed an AUC of 0.70; because the observed discriminatory effects were generally smaller, the study may have had limited power to detect modest discrimination.

## 5. Conclusions

Anthropometric and body composition measures showed limited ability to discriminate CONUT-defined nutritional risk in hospitalized older adults and should not be considered substitutes for CONUT-based classification. Among the evaluated measures, fat mass percentage showed the most robust association with CONUT-defined nutritional risk. More complex derived anthropometric indices did not provide additional discriminatory value in this population. Further studies involving larger and more diverse populations, including community-dwelling older adults, are warranted to confirm these findings and assess their generalizability beyond the hospital setting.

## Figures and Tables

**Table 1:** Definitions and formulas of the anthropometric and adiposity indices used in the study.

Index	Formula	Original Reference
Body mass index (BMI)	body weight (kg)/height squared (m^2^)	[19]
Waist-to-hip ratio (WHR)	waist circumference (cm)/hip circumference (cm)	[14]
Waist-to-height ratio (WHtR)	waist circumference (cm)/height (cm)	[20]
Body adiposity index (BAI)	hip circumference (cm)/[height (m)]^1.5^ − 18	[21]
A body shape index (ABSI)	Waist circumference (m)/((BMI)^2/3^) × [height (m)]^1/2^	[22]
Body roundness index (BRI)	= 364.2 − 365.5 × √{1 − ((waist circumference/2π)^2^/[0.5 × height]^2^)}	[23]
Abdominal volume index (AVI)	[2 × (waist circumference in cm)^2^ + 0.7 × (waist circumference in cm − hip circumference in cm)^2^]/1000	[24]
Visceral adiposity index (VAI)	For women: (waist circumference [cm]/(36.58 + 1.89 × BMI)) × (triglycerides [mmol/L]/0.81) × (1.52/HDL cholesterol [mmol/L])	[25]
	For men: (waist circumference [cm]/(39.68 + 1.88 × BMI)) × (triglycerides [mmol/L]/1.03) × (1.31/HDL cholesterol [mmol/L])	
Weight-adjusted waist index (WWI)	waist circumference (cm)/√body weight (kg)	[26]

**Table 2:** Calculation and interpretation of the Controlling Nutritional Status (CONUT) score [6].

Parameter	The Controlling Nutritional Status			
Serum albumin (g/dL)	≥3.50	3.0–3.49	2.5–2.99	<2.5
Albumin score	0	2	4	6
Total cholesterol (mg/dL)	>180	140–180	100–139	<100
Cholesterol score	0	1	2	3
Total lymphocyte count (cells/mm^3^)	>1600	1200–1599	800–1199	<800
Lymphocyte score	0	1	2	3
Total CONUT score	0–1	2–4	5–8	9–12
CONUT category	Normal	Mild nutritional risk	Moderate nutritional risk	Severe nutritional risk

**Table 3:** Demographic and clinical characteristics (*N* = 164).

	Total Group (*N* = 164) ^1^	CONUT-Normal Group (*N* = 93) ^2^	CONUT-Defined Nutritional Risk Group (*N* = 71) ^2^	*p* Value ^3^
Demographic characteristics				
Age (years)	79.03 (6.31)	78.11 (6.19)	80.23 (6.32)	0.034
Women, *n* (%)	118 (72.0)	69 (74.2)	49 (69.0)	0.5
Clinical characteristics				
Heart failure, *n* (%)	61 (37.2)	35 (37.6)	26 (36.6)	0.9
Coronary artery disease, *n* (%)	60 (36.6)	38 (40.9)	22 (31.0)	0.2
Previous myocardial infarction, *n* (%)	14 (8.5)	10 (10.8)	4 (5.6)	0.2
Atrial fibrillation, *n* (%)	34 (20.7)	14 (15.1)	20 (28.2)	0.040
Arterial hypertension, *n* (%)	134 (81.7)	82 (88.2)	52 (73.2)	0.014
Previous stroke, *n* (%)	23 (14.0)	12 (12.9)	11 (15.5)	0.6
Dementia, *n* (%)	34 (20.7)	22 (23.7)	12 (16.9)	0.3
Chronic obstructive pulmonary disease, *n* (%)	14 (8.5)	9 (9.7)	5 (7.0)	0.5
Chronic kidney disease, stage 1–3, *n* (%)	23 (14.0)	12 (12.9)	11 (15.5)	0.6
Diabetes mellitus, *n* (%)	55 (33.5)	38 (40.9)	17 (23.9)	0.023
Anemia, *n* (%)	40 (24.4)	12 (12.9)	28 (39.4)	<0.001
Statin use, *n* (%)	40 (24.4)	24 (25.8%)	16 (22.5%)	0.6
Laboratory parameters				
Albumin, g/dL	3.6 (3.4–3.9)	3.8 (3.6–3.9)	3.4 (3.2–3.5)	<0.001
Total lymphocyte count (cells/mm^3^)	2208 (1833–2760)	2346 (1968–2993)	2010 (1584–2370)	<0.001
Total cholesterol (mmol/L)	4.71 (4.01–5.56)	5.05 (4.38–5.96)	4.07 (3.44–5.21)	<0.001
LDL cholesterol (mmol/L)	2.75 (2.14–3.52)	2.93 (2.35–3.66)	2.29 (1.64–2.98)	<0.001
HDL cholesterol (mmol/L)	1.41 (1.11–1.74)	1.42 (1.19–1.68)	1.40 (1.06–1.79)	0.5
Triglycerides (mmol/L)	1.19 (0.95–1.55)	1.37 (1.01–1.73)	1.06 (0.87–1.28)	<0.001
C-reactive protein (mg/L)	2.10 (0.60–5.30)	1.65 (0.50–4.15)	2.50 (1.00–6.30)	0.015
eGFR (mL/min/1.73 m^2^)	71.93 (20.38)	70.80 (20.07)	73.35 (20.82)	0.4

^1^ The number of available observations varied for variables with missing data: triglycerides, *n* = 162; C-reactive protein, *n* = 163; and eGFR, *n* = 151. All other variables presented in the table were available for the full study population (*N* = 164). ^2^ Mean (SD); Median (Q1, Q3); *n* (%). ^3^ Welch Two Sample *t*-test; Wilcoxon rank sum test; Fisher’s exact test; Wilcoxon rank sum exact test. Abbreviations: CRP, C-reactive protein; eGFR, estimated glomerular filtration rate; HDL, high-density lipoprotein; LDL, low-density lipoprotein; TG, triglycerides.

**Table 4:** Anthropometric measurements, body composition parameters, and anthropometric indices according to CONUT category (*N* = 164).

	CONUT-Normal Group (*N* = 93) ^1^	CONUT-Defined Nutritional Risk Group (*N* = 71) ^1^	*p* Value ^2^
Anthropometric measurements			
Body weight, kg	69.20 (61.20–79.40)	64.00 (54.30–74.70)	0.014
Height, cm	156.00 (152–160)	153.50 (149–162.50)	0.5
Hip circumference, cm	101.90 (12.34)	98.97 (14.81)	0.2
Waist circumference, cm	105.00 (99.00–114.00)	101.00 (95.00–109.00)	0.013
Mid-upper arm circumference, cm	29.37 (4.06)	27.27 (4.25)	0.002
Calf circumference, cm	35.35 (4.05)	33.82 (3.70)	0.012
Body composition parameters			
Fat mass, kg	23.90 (19.30–31.60)	20.10 (13.30–26.40)	0.001
Fat mass, %	36.05 (29.20–42.85)	30.55 (24.10–37.20)	<0.001
Fat-free mass, kg	43.75 (39.40–50.70)	41.55 (38.30–50.60)	0.5
Muscle mass, kg	41.55 (37.40–48.10)	39.45 (36.30–48.00)	0.5
Derived anthropometric indices			
BMI, kg/m^2^	27.70 (25.50–31.70)	25.90 (22.80–29.80)	0.009
WHR	0.95 (0.06)	0.96 (0.07)	0.3
WHtR	0.65 (0.08)	0.63 (0.10)	0.2
AVI	20.40 (17.46–24.20)	19.21 (15.84–23.14)	0.12
ABSI	0.09 (0.01)	0.09 (0.01)	0.028
BRI	6.54 (5.12–7.96)	6.19 (4.74–7.79)	0.2
BAI, %	36.40 (31.34–41.16)	34.37 (29.34–38.59)	0.042
VAI	1.67 (1.23–2.63)	1.48 (0.90–2.08)	0.059
WWI	12.81 (0.83)	12.78 (0.90)	0.8

^1^ Mean (SD); Median (Q1, Q3); *n* (%). ^2^ Welch Two Sample *t*-test; Wilcoxon rank sum test; Fisher’s exact test; Wilcoxon rank sum exact test. Abbreviations: BMI, body mass index; WHR, waist-to-hip ratio; WHtR, waist-to-height ratio; BAI, body adiposity index; ABSI, a body shape index; BRI, body roundness index; AVI, abdominal volume index; VAI, visceral adiposity index; WWI, weight-adjusted waist index.

**Table 5:** Correlations of anthropometric measurements, body composition parameters, and anthropometric indices with the CONUT score (*N* = 164).

Variable		Spearman’s ρ	*p* Value
Age, years	CONUT	0.15	0.050
Body weight		−0.21	0.008
Waist circumference		−0.12	0.124
Mid-upper arm circumference		−0.19	0.017
Calf circumference		−0.17	0.034
Fat mass, kg		−0.25	0.002
Fat mass, %		−0.25	0.001
Muscle mass, kg		−0.08	0.330
BMI		−0.18	0.021
WHR		0.05	0.514
WHtR		−0.10	0.223
BAI		−0.11	0.164
ABSI		0.15	0.057
BRI		−0.10	0.223
AVI		−0.12	0.120
VAI		−0.13	0.097
WWI		0.08	0.326

ρ, Spearman’s rank correlation coefficient. Abbreviations: BMI, body mass index; WHR, waist-to-hip ratio; WHtR, waist-to-height ratio; BAI, body adiposity index; ABSI, a body shape index; BRI, body roundness index; AVI, abdominal volume index; VAI, visceral adiposity index; WWI, weight-adjusted waist index.

**Table 6:** Associations of anthropometric measurements, body composition parameters, and anthropometric indices with CONUT-defined nutritional risk in multivariable logistic regression models.

Variable	Model 1 OR (95% CI)	*p* Value	q	Model 2 OR (95% CI)	*p* Value	q	Model 3 OR (95% CI)	*p* Value	q	Model 4 OR (95% CI)	*p* Value	q
Body weight	0.981 (0.958–1.005)	0.1237	0.247	0.982 (0.958–1.006)	0.1417	0.283	0.982 (0.958–1.007)	0.1512	0.302	0.982 (0.958–1.007)	0.1563	0.313
Waist circumference	0.987 (0.964–1.012)	0.3112	0.453	0.988 (0.964–1.013)	0.3412	0.496	0.985 (0.961–1.01)	0.2447	0.392	0.985 (0.961–1.011)	0.2521	0.403
Mid-upper arm circumference	0.895 (0.822–0.973)	0.0094	0.054	0.895 (0.822–0.975)	0.0112	0.062	0.901 (0.827–0.982)	0.0176	0.094	0.9 (0.825–0.982)	0.0177	0.094
Calf circumference	0.911 (0.832–0.998)	0.0443	0.177	0.912 (0.833–0.999)	0.0481	0.192	0.921 (0.84–1.01)	0.0806	0.258	0.921 (0.84–1.011)	0.0821	0.263
Fat mass, kg	0.955 (0.922–0.989)	0.0101	0.054	0.955 (0.922–0.99)	0.0117	0.062	0.956 (0.922–0.991)	0.0136	0.094	0.955 (0.921–0.991)	0.0136	0.094
Fat mass, %	0.931 (0.891–0.973)	0.0014	0.022	0.931 (0.891–0.973)	0.0016	0.026	0.929 (0.888–0.972)	0.0015	0.024	0.928 (0.887–0.972)	0.0015	0.024
Muscle mass, kg	1.02 (0.959–1.084)	0.528	0.580	1.022 (0.961–1.087)	0.4878	0.557	1.023 (0.96–1.091)	0.4801	0.549	1.024 (0.96–1.092)	0.4688	0.536
BMI	0.946 (0.889–1.007)	0.0801	0.247	0.947 (0.89–1.008)	0.0882	0.261	0.948 (0.889–1.01)	0.0998	0.262	0.948 (0.889–1.011)	0.1023	0.265
WHR ^1^	1.167 (0.708–1.924)	0.5442	0.453	1.173 (0.711–1.935)	0.5324	0.496	1.026 (0.615–1.712)	0.9223	0.381	1.029 (0.616–1.719)	0.9144	0.389
WHtR	0.137 (0.003–5.651)	0.2951	0.580	0.147 (0.004–6.065)	0.3121	0.568	0.086 (0.002–4.122)	0.2143	0.922	0.088 (0.002–4.227)	0.2186	0.914
BAI	0.96 (0.912–1.01)	0.1151	0.247	0.96 (0.912–1.011)	0.1197	0.274	0.957 (0.907–1.011)	0.1147	0.262	0.958 (0.907–1.011)	0.1159	0.265
ABSI ^2^	1.685 (0.909–3.123)	0.0973	0.247	1.685 (0.908–3.127)	0.0979	0.261	1.409 (0.742–2.674)	0.2945	0.428	1.412 (0.744–2.683)	0.2914	0.424
BRI	0.941 (0.812–1.091)	0.4186	0.538	0.944 (0.814–1.094)	0.4408	0.557	0.927 (0.795–1.081)	0.3352	0.447	0.928 (0.796–1.082)	0.3415	0.455
AVI	0.977 (0.921–1.036)	0.4375	0.538	0.979 (0.922–1.039)	0.4775	0.557	0.973 (0.916–1.034)	0.3766	0.464	0.973 (0.916–1.035)	0.3876	0.477
VAI	0.872 (0.721–1.055)	0.1578	0.281	0.874 (0.723–1.056)	0.1636	0.291	0.816 (0.658–1.012)	0.0642	0.257	0.817 (0.659–1.013)	0.0659	0.263
WWI	0.973 (0.582–1.627)	0.9175	0.918	0.97 (0.58–1.622)	0.9062	0.906	0.942 (0.541–1.641)	0.8334	0.889	0.941 (0.54–1.639)	0.8303	0.886

**Model 1:** adjusted for age and sex. **Model 2:** adjusted for age, sex, and statin use. **Model 3:** adjusted for age, sex, and C-reactive protein. **Model 4:** adjusted for age, sex, statin use, and C-reactive protein. Each anthropometric measurement, body composition parameter, and derived anthropometric index was entered into a separate logistic regression model. **Abbreviations:** BMI, body mass index; WHR, waist-to-hip ratio; WHtR, waist-to-height ratio; BAI, body adiposity index; ABSI, a body shape index; BRI, body roundness index; AVI, abdominal volume index; VAI, visceral adiposity index; WWI, weight-adjusted waist index. ^1^ For WHR, odds ratios are expressed per 0.1-unit increase. ^2^ For ABSI, odds ratios are expressed per 0.01-unit increase. q, false discovery rate (FDR)-adjusted *p* value using the Benjamini–Hochberg procedure.

**Table 7:** Discriminative performance and exploratory cut-off values of anthropometric measurements, body composition parameters, and anthropometric indices for identifying CONUT-defined nutritional risk.

Parameters	Parameter Cut-Off	AUC (95% CI)	Bootstrap-Validated *C*-Index (95% CI)	Sensitivity	Specificity	PPV	NPV
Body weight	65.3	0.612 (0.523–0.701)	0.611 (0.519–0.713)	0.59	0.66	0.57	0.68
Waist circumference	90	0.569 (0.478–0.66)	0.569 (0.475–0.643)	0.34	0.85	0.63	0.63
Mid-upper arm circumference	29	0.638 (0.552–0.723)	0.642 (0.556–0.731)	0.73	0.51	0.53	0.71
Calf circumference	34.5	0.609 (0.522–0.696)	0.61 (0.525–0.702)	0.66	0.57	0.54	0.69
Fat mass, kg	21.4	0.647 (0.56–0.734)	0.641 (0.563–0.719)	0.63	0.64	0.57	0.69
Fat mass, %	38.5	0.655 (0.57–0.741)	0.651 (0.573–0.734)	0.86	0.42	0.53	0.80
Muscle mass, kg	52.7	0.470 (0.378–0.563)	0.407 (0.314–0.5)	0.19	0.92	0.65	0.60
BMI	25	0.620 (0.531–0.709)	0.616 (0.527–0.701)	0.47	0.80	0.64	0.66
WHR	0.923	0.552 (0.463–0.641)	0.547 (0.468–0.615)	0.76	0.39	0.49	0.68
WHtR	0.582	0.564 (0.473–0.654)	0.56 (0.473–0.63)	0.37	0.82	0.61	0.63
BAI	38.692	0.593 (0.504–0.681)	0.595 (0.512–0.677)	0.78	0.41	0.50	0.70
ABSI	0.09	0.599 (0.51–0.687)	0.596 (0.504–0.687)	0.48	0.74	0.59	0.65
BRI	5.034	0.564 (0.473–0.654)	0.557 (0.475–0.644)	0.37	0.82	0.61	0.63
AVI	17.298	0.57 (0.479–0.662)	0.574 (0.487–0.664)	0.39	0.81	0.61	0.64
VAI	1.884	0.587 (0.498–0.675)	0.588 (0.499–0.688)	0.73	0.44	0.51	0.68
WWI	12.173	0.496 (0.406–0.587)	0.458 (0.389–0.506)	0.30	0.77	0.50	0.59

**Abbreviations:** AUC, area under the curve; CI, confidence interval; *C*-index, concordance index; NPV, negative predictive value; PPV, positive predictive value; ABSI, a body shape index; AVI, abdominal volume index; BAI, body adiposity index; BMI, body mass index; BRI, body roundness index; CONUT, Controlling Nutritional Status; VAI, visceral adiposity index; WHR, waist-to-hip ratio; WHtR, waist-to-height ratio; WWI, weight-adjusted waist index.

## Data Availability

The data presented in this study are available from the corresponding author upon reasonable written request. The data are not publicly available due to privacy and ethical considerations related to the protection of study participants.

## Abbreviations

The following abbreviations are used in this manuscript:

ABSI	A body shape index
AUC	Area under the curve
AVI	Abdominal volume index
BAI	Body adiposity index
BIA	Bioelectrical impedance analysis
BIVA	Bioelectrical impedance vector analysis
BMI	Body mass index
BRI	Body roundness index
CI	Confidence interval
CKD	Chronic kidney disease
CONUT	Controlling Nutritional Status
CRP	C-reactive protein
CT	Computed tomography
DXA	Dual-energy X-ray absorptiometry
eGFR	Estimated glomerular filtration rate
EWGSOP2	European Working Group on Sarcopenia in Older People 2
GLIM	Global Leadership Initiative on Malnutrition
GNRI	Geriatric Nutritional Risk Index
HDL	High-density lipoprotein
LDL	Low-density lipoprotein
MNA	Mini Nutritional Assessment
MRI	Magnetic resonance imaging
NPV	Negative predictive value
OR	Odds ratio
PPV	Positive predictive value
ROC	Receiver operating characteristic
SD	Standard deviation
VAI	Visceral adiposity index
WHR	Waist-to-hip ratio
WHtR	Waist-to-height ratio
WWI	Weight-adjusted waist index
